# Fear of COVID-19 and Vaccine Hesitancy among Pregnant Women in Poland: A Cross-Sectional Study

**DOI:** 10.3390/vaccines10101700

**Published:** 2022-10-11

**Authors:** Kinga Janik, Kinga Nietupska, Grazyna Iwanowicz-Palus, Mateusz Cybulski

**Affiliations:** 1 Department of Integrated Medical Care, Faculty of Health Sciences, Medical University of Bialystok, M. Skłodowskiej-Curie 7A Str., 15-096 Bialystok, Poland; 2 Department of Obstetrics and Gynecology, Independent Public Healthcare Center in Sokolka, Sikorskiego 40 Str., 16-100 Sokolka, Poland; 3 Department of Development in Midwifery, Faculty of Health Sciences, Medical University of Lublin, Staszica 4/6 Str., 20-081 Lublin, Poland

**Keywords:** anxiety, attitudes, COVID-19, fear, pregnant, SARS-CoV-2, vaccines

## Abstract

Introduction: Pregnant women are particularly vulnerable to anxiety and stress, and the COVID-19 pandemic has definitely contributed to anxiety in this group. Researchers continue their work on COVID-19 vaccine formulations to reduce the spread of the SARS-CoV-2 virus and minimise the impact of the pandemic. Despite the increased prevalence and severity of anxiety among pregnant women during the COVID-19 pandemic, their attitudes towards COVID-19 vaccine vary. The aim of this study was to assess the levels of anxiety experienced by pregnant women due to COVID-19 and their attitudes to vaccination. Materials and methods: A total of 595 women voluntarily participated in the study. The respondents were divided into two groups: the study group (*n* = 288), which consisted of women who were pregnant at the time of the survey, and the control group (*n* = 307), which included women of reproductive age (18–49 years). The study used a diagnostic survey method with a web-based questionnaire consisting of the author’s survey questionnaire and the following standardised tools: the Scale to Measure the Perception of SARS-CoV-2 Vaccines Acceptance (VAC-COVID-19 SCALE), the Fear of COVID-19 Scale (FCV-19S), the Drivers of COVID-19 Vaccination Acceptance Scale (DrVac-COVID19S) and the Coronavirus Anxiety Scale (CAS). Results: The level of COVID-related anxiety differed depending on the tool used. Mild anxiety was reported for CAS, while FCV-19S showed its moderate levels. Both pregnant women and women of reproductive age showed high scores in VAC-COVID-19 and DrVac-COVID19S. The mean VAC-COVID-19 scores were 41.44 in the study group and 44.26 in the control group, and the mean DrVac-COVID19S scores were 51.25 in pregnant women and 55.85 in women of reproductive age. This indicates a high level of acceptance of and positive attitudes toward vaccinations. Conclusions: Pregnant women showed moderate coronavirus anxiety. Women in both the study group and the control group showed mostly positive attitudes towards COVID-19 vaccination.

## 1. Introduction

COVID-19 infection, responsible for severe acute respiratory distress syndrome, was declared a global pandemic by the World Health Organisation (WHO) in March 2020 [1]. The pandemic has caused serious conditions and death among millions of people worldwide [1,2]. Various COVID-19 vaccination studies have shown over 90% efficacy in preventing severe COVID-19 disease and mortality [3,4,5]. Worldwide, more than 63% of the population is fully vaccinated, and extensive vaccination programmes are underway. As of 4 March 2020, 6,213,262 cases of COVID-19 were reported in Poland, of which 5,335,955 recovered and 117,252 were fatal [6]. By 12 September 2022, 56,332,553 vaccinations had been administered in Poland. The number of fully vaccinated people is estimated at 22,551,992 [7].

Pregnancy is a special period in a woman’s life with increased levels of anxiety, stress and concern about their unborn child’s and their own health. Additional negative factors include a relevant obstetric history, previous miscarriages or perinatal complications [8]. Mental health is an important factor influencing maternal wellbeing and foetal development [9].

Approximately 15% of all pregnant women experience emotional changes that increase the risk of anxiety and depression, which can in turn adversely affect their health and their developing foetuses [10]. Children of mothers who exhibit high levels of anxiety and stress during pregnancy may experience emotional, behavioural and cognitive problems and are at higher risk of neurodevelopmental disorders [11]. There are literature reports indicating a significantly higher prevalence of anxiety and depressive symptoms in pregnant women during the COVID-19 pandemic than before the pandemic [12,13]; however, their exact prevalence is currently unknown [14]. However, studies to date on fear of COVID-19 have shown that women were more frightened and concerned about COVID-19 than men [15,16]. Fears of health consequences had a particularly negative impact on their mental status [17]. Additionally, the age under 45 has proved to be a significant risk factor for impairment of both cognitive functions and mental health [15,18].

Despite the higher prevalence and severity of anxiety among pregnant women during the COVID-19 pandemic, their attitudes towards COVID-19 vaccination vary. Almost half of the women surveyed said they would vaccinate if it were recommended for pregnant women. An equally large number of pregnant women refused to take the vaccine. Insufficient data on the safety of the COVID-19 vaccine in pregnancy and reports on possible foetal toxicity are the most common reasons for refusal [19]. However, data collected in the scientific literature suggest that the benefits of COVID-19 vaccination outweigh any known or potential risks during pregnancy [20]. According to the Centers for Disease Control and Prevention (CDC), there is no evidence to suggest that vaccination may cause harm during pregnancy or that there are any safety concerns regarding pregnancy or the impact on the newborn child [20,21,22]. The WHO approved COVID-19 vaccine for pregnant women soon after its introduction. Despite the widespread availability of COVID-19 vaccine, limited global data are available on pregnant women’s knowledge, attitudes and practices related to the vaccine [23], as few population-based prospective studies on COVID-19 vaccine have been conducted among pregnant women to date. 

By definition, COVID-19 causes significant fear and anxiety among the population. From our observation, there are insufficient data on the severity of anxiety resulting from the COVID-19 pandemic among pregnant women in Poland. Little is known about the fear of COVID-19 or its effect on maternal psychological distress or birth outcomes. This is an important gap because it limits the ability of healthcare providers and health systems to anticipate and rapidly respond to the needs of pregnant individuals when epidemic and pandemic infectious diseases arise. There are also insufficient data on opinions regarding COVID-19 vaccination among pregnant women. Despite the importance of vaccination to COVID-19 prevention and management as well as the low rate of vaccination among pregnant women in the world, there are limited data about pregnant women’s acceptance of COVID-19 vaccine. Consequently, the present study was conducted to narrow this gap. Therefore, the aim of this study was to analyse and assess the prevalence of COVID-19 anxiety symptoms and to investigate the attitudes towards COVID-19 vaccination among pregnant women. We assumed that the severity of anxiety disorder symptoms among pregnant women in Poland would oscillate at a moderate level, while their attitudes towards vaccination would vary, with a similar proportion of supporters and opponents. In addition, the analysis of pregnant women’s attitudes towards vaccination may be useful for health education of patients and help prevent COVID-19 during pregnancy.

## 2. Materials and Methods

### 2.1. Participants

A minimum sample size of at least 384 enrolled individuals would have been required to investigate the selected variables in pregnant women in Poland (i.e., 211,022 pregnant women in Poland in 2021). The sample was calculated by a sample size calculator, based on the reference population, assuming a response proportion of 50%, a 95% confidence level, and a 5% margin of error.

Finally, the study included 288 women at different stages of pregnancy as the study group and 307 women of reproductive age as the control group. A total of 595 women participated in the study. The sample was not representative, because the number of births in Poland has been constantly decreasing in recent years.

#### 2.1.1. Study Group

The age of respondents ranged from 19 to 42 years. Most of them were married (82%). Almost 47% had a university education. Less than 39% declared rural areas as their place of residence, with the remaining women residing in cities throughout Poland. Over 62% of respondents described their socioeconomic status as “good”. 

#### 2.1.2. Control Group

The age criterion ranged from 18 to 49 years. The mean age in the group was 31 years. The majority of respondents were married (50%). Almost 51% had a university education. Less than 29% of the respondents declared rural areas as their place of residence; the remaining women were city dwellers from all over Poland. A total of 62% of respondents described their socioeconomic status as “good”. 

Detailed socio-demographic characteristics are shown in Table 1. 

### 2.2. Study Design and Data Collection

An original author’s questionnaire, designed for the purpose of the present study, was used. It included questions on socio-demographic characteristics and detailed questions on past COVID-19 infection, vaccination and opinion on COVID-19 vaccine. In addition to questions on socio-demographic characteristics, the questionnaire contained eight closed-ended questions, four single-choice and four multiple-choice questions. Standardised survey instruments, which are discussed below, were also used. 

The survey was conducted between 5 February 2022 and 20 April 2022. A link to the dedicated questionnaire on the Webankieta platform was posted on social media in discussion groups dedicated to pregnant women (study group) and discussion groups aimed at young mothers and women of reproductive age (control group). Interested women were able to voluntarily participate in the online survey. The responses were recorded on the platform, then downloaded as raw data and statistically analysed using dedicated specialised software. 

Participation in the anonymous study was voluntary and tantamount to consenting to the use of the acquired data for scientific purposes. Ongoing pregnancy was the only criterion for inclusion in the study group. Each participant could withdraw from the study at any time. Age between 18 and 49 years was the criterion for inclusion in the control group. 

There were 1044 visits on the platform, which yielded 57% of fully completed questionnaires. There were 113 incomplete questionnaires. 

### 2.3. Measures

The following standardised psychometric scales were used in the study: The Fear of COVID-19 Scale (FCV-19S), Coronavirus Anxiety Scale (CAS), The Drivers of COVID-19 Vaccination Acceptance Scale (DrVac-COVID19S) and the Scale to Measure the Perception of SARS-CoV-2 Vaccines Acceptance (The VAC-COVID-19 Scale).

The Fear of COVID-19 Scale (FCV-19S) was developed to measure anxiety and fear of COVID-19. FCV-19S is a simple seven-item self-administered scale developed by Ahorsu et al. [24]. Answers included “strongly disagree,” “disagree,” “neither agree nor disagree,” “agree”, and “strongly agree”. The minimum score possible for each question is 1 (strongly disagree), and the maximum is 5 (strongly agree). The total score is calculated by adding up each item score (ranging from 7 to 35). The higher the score, the greater the fear of COVID-19 [25]. FCV-19S has been translated and validated in a number of countries, including Poland. This tool obtained high reliabilities in the Polish validation study, with Cronbach’s α of 0.85–0.89 [26]. 

The Coronavirus Anxiety Scale (CAS) is a brief self-reported mental health screener of dysfunctional anxiety associated with the coronavirus crisis, which consists of five items related to a variety of physical and mental ailments that appear in response to news or thoughts about the coronavirus. Each item contains answers from 0 (“not at all”) to 4 (“nearly every day over the last 2 weeks”) [27]. Cronbach’s α and McDonald’s ω coefficients for the Polish version of CAS were α = 0.93 and ω = 0.93, respectively [28].

The VAC-COVID-19 scale is a valid and reliable instrument of public health to measure perceptions of SARS-CoV-2 vaccine acceptance. This scale can be very useful to determine the reasons why different populations adhere or not to the vaccination, in order to help propose adequate and effective strategies to advance vaccination coverage rates. The VAC-COVID-19 scale is a simple eleven-item self-administered scale developed by Mejia et al. [29]. There are two groups of factors: positive (reasons for receiving vaccination) and negative (reasons for not receiving vaccination). Each item had five possible Likert-type responses: strongly disagree, disagree, neither disagree nor agree, agree, and strongly agree. The minimum score possible for each question is 1 (strongly disagree), and the maximum is 5 (strongly agree). The reverse scoring applies to the second (negative) group of factors. The total score is calculated by adding up each item score (ranging from 11 to 55). The higher the score, the more positive attitudes towards COVID-19 vaccinations. Cronbach’s α coefficient for this tool was α = 0.831 [29].

The DrVac-COVID19S was adapted from the MoVac-Flu Scale [30]. The major difference between the DrVac-COVID19S and the MoVac-Flu Scale is that the MoVac-Flu Scale uses the word flu, and the DrVac-COVID19S uses the word COVID-19. The DrVac-COVID19S contains 12 items, where nine items are positively worded (items 1 to 6, 8, 9, and 12) and three items are negatively worded (items 7, 10, and 11). Therefore, the DrVac-COVID19S shares the same model of CME as the MoVac-Flu Scale in assessing an individual’s values, impacts, knowledge, and autonomy traits. The four traits can help healthcare providers and researchers to understand how an individual cares about the purpose of COVID-19 vaccination uptake (values); believes in the effects of COVID-19 vaccination uptake in preventing COVID-19 infection (impacts); has knowledge regarding the COVID-19 vaccination uptake (knowledge); and is confident and has control in obtaining COVID-19 vaccination if the individual wants to (autonomy). Moreover, the 12 items comprise four traits corresponding to the CME model: items 3 (“It is important that I get the COVID-19 jab”), 6 (“The COVID-19 jab plays an important role in protecting my life and that of others”), and 8 (“The contribution of the COVID-19 jab to my health and well-being is very important”) comprise values; items 1 (“Vaccination is a very effective way to protect me against COVID-19”), 4 (“Vaccination greatly reduces my risk of catching COVID-19”), and 12 (“Getting the COVID-19 jab has a positive influence on my health”) comprise impacts; items 2 (“I know very well how vaccination protects me from COVID-19”), 5 (“I understand how the flu jab helps my body fight the COVID-19 virus”), and 10 ("How the COVID-19 jab works to protect my health is a mystery to me”) comprise knowledge; and items 7 (“I feel under pressure to get the COVID-19 jab”), 9 ("I can choose whether to get a COVID-19 jab or not”), and 11 (“I get the COVID-19 jab only because I am required to do so”) comprise autonomy. All the items are rated using a seven-point Likert scale. After reverse coding, the negatively worded items (i.e., scoring for these items is from 1 (strongly agree) to 7 (strongly disagree)), a higher score in the DrVac-COVID19S indicates a higher level of COVID-19 vaccine acceptance. Cronbach’s α in this scale was 0.86 [30].

### 2.4. Procedure and Ethical Considerations

The study was conducted in accordance with the recommendations of, and was reviewed and approved by, the Ethics Committee of the Medical University of Bialystok (No. APK.002.55.2022). All participants gave a written informed consent in accordance with the Declaration of Helsinki.

### 2.5. Statistical Analysis

Statistica 13.3 (StatSoft Polska, Krakow, Poland) was used for statistical analysis. The analysed variables were of nominal, interval, or ordinal nature. The chi-square test was used to assess relationships between nominal characteristics. For interval variables, Spearman’s rank correlation coefficient was used. The Mann–Whitney U test was performed to compare two groups. Kruskal–Wallis test with post hoc tests were used for more than two groups. The level of statistical significance was set at *p* < 0.05 for each test. 

## 3. Results

Table 2 shows the analysis of questions from the author’s survey questionnaire. There was a significantly lower percentage of vaccinated women in the study group than in the control group. Additionally, the number of vaccinated and unvaccinated pregnant women was similar in the study group.

The analysis showed that the incidence rate of COVID-19 was significantly lower among pregnant women than in the control group. We did not investigate whether COVID-19 occurred before or after vaccination. Pfizer vaccine was the most common choice, while the Johnson & Johnson vaccine was the least common choice in both the study group and control group.

Pain at the injection site was the most common symptom reported after receiving the vaccine in the study group (>50%). The same symptom occurred in almost ¾ of respondents in the control group. No vaccine-induced symptoms occurred in ⅓ of the pregnant women. The same was reported by only 18% of the control group. 

Both groups were asked about their opinions on vaccination of pregnant women. According to 25% of the study group and 42% of the control group, vaccination was safe and necessary, while 10% of pregnant women and 8% of women of reproductive age believed that the vaccine was dangerous. The opinion that vaccination is contraindicated in pregnancy was shared by a similar percentage of respondents in the study and control groups, i.e., 5% and 7%, respectively. 

The respondents were asked the question “What do you think are the effects of COVID-19 vaccination?”. Less than half of the study and control group chose the answer “acquisition of specific immunity against COVID-19 by both mother and child”, whereas ⅕ of the pregnant women believed that the vaccine causes defects in the foetus. This opinion was shared by only 12% of women in the control group. The answer “acquisition of specific immunity against COVID-19 by the child” was least common (only 3% in both groups).

A summary of COVID-19 and vaccination rates among the respondents is shown in Table 3. In the group of pregnant women, less than 12% of vaccine recipients contracted COVID-19. Of the vaccinated pregnant women, 88% avoided COVID-19. No statistically significant differences were found between vaccine coverage and COVID-19 morbidity in the control group. We did not investigate whether the infection occurred before or after receiving the vaccine. 

Table 4 shows the descriptive statistics of the standardised scales used in the study. We found statistically significant differences between the groups for CAS (*p* = 0.025), DrVac-COVID19S (*p* = 0.00) or VAC-COVID-19 (*p* = 0.00). From the results, it can be seen that pregnant women scored significantly lower. No statistically significant differences were found in FCV-19S. Furthermore, both pregnant women and controls showed positive attitudes towards COVID-19 vaccination. The mean VAC-COVID-19 score was 44.26 in the control group and 41.44 in the study group. These results correspond with DrVac-COVID19S scores. Here, the mean score was 51.25 for pregnant women and 55.85 for women of reproductive age, which also indicates a high level of vaccine acceptance and positive attitudes towards it. The mean CAS score was 0.61 in the pregnant group and 1.03 in the control group, respectively, suggesting a low level of anxiety associated with COVID-19. Other results on the severity of anxiety and fear of coronavirus were obtained using the FCV-19S. The mean score oscillated around 15 out of a possible 35, indicating moderate anxiety. 

Table 5 summarises scores obtained from pregnant women using the standardised tools by trimester of pregnancy. The analysis shows that the fear of coronavirus increased with increasing pregnancy time. Pregnant women in the first trimester had lower (12.34) FCV-19S scores than pregnant women in the second (14.70) and third trimester (15.16) of pregnancy. In the other scales, there were no significant differences in scores depending on the trimester of pregnancy. The level of anxiety in the group of pregnant women could be described as moderate and increasing with the approaching birth.

Table 6 shows the results of inter-scale correlation by groups. The strongest correlation was found between VAC-COVID-19 and DrVac-COVID-19S. The Spearman coefficient was 0.722 for the control group and 0.753 for the study group, indicating a strong positive relationship. There was also a positive correlation between CAS and FCV-19S, but less significant. The Spearman coefficient was 0.377 for the control group and 0.364 for the study group. In the remaining scales, the correlations were statistically significant, but due to the low number of respondents in the groups, the results cannot be projected to the entire population.

## 4. Discussion

In the general population, based on the study by Barchielli et al. [15], it was shown that the fear of the possible consequences of COVID-19 vaccinations was more often reported by older adults, while the fear of the disease, its consequences and the probability of isolation was most often reported by young adults compared to elderly people. This finding may be related to greater awareness of COVID-19 and preventive measures among older adults [31], which may explain less concern. At the same time, the importance of social ties among young people should also be taken into account, explaining why young adults experienced isolation with greater anxiety and negative effects on mental health [32,33]. In line with the results of previous studies [16,17], women reported greater fear of the disease and its consequences, but not in the older group. Again, this can be explained by less concern among the older adults, regardless of sex.

The presented paper is one of the few to investigate the level of COVID-19 pandemic-related anxiety among pregnant women and their attitudes towards COVID-19 vaccine using standardised, dedicated survey tools. The study found different levels of COVID-19 anxiety in pregnant women depending on the standardised scale used. 

Several papers have documented that SARS-CoV-2 virus infection during pregnancy is closely associated with a severe course of the disease and many adverse obstetric complications [34,35,36].

Lin et al. conducted a study to assess COVID-19 anxiety using FCV-19S in 11 countries. Their results varied from country to country. Iranians showed the highest level of anxiety, with a mean score of 3.92. Respondents from New Zealand showed the lowest anxiety, with a mean score of 2.02 [37]. In our study, the mean anxiety scores obtained with the same standardised tool were 15.21 in the control group and 14.59 in the study group. Contrarily, anxiety levels in pregnant women with SARS-CoV-2 were low at the end of the pandemic in the UK, which was due to increased available clinical information and reassurance via social media, health-care professionals and primary care; however, the sample size in this study was too small to achieve a statistically valid result [38]. Another study assessed the impact of COVID-19-related anxiety and fear on perinatal depression among pregnant women in Italy. The mean FCV-19S and CAS scores were 15.0 and 1.7, respectively [39]. In our study, the scores of pregnant women were similar and were 14.6 in FCV-19S and 0.6 in CAS, respectively. Thus, it can be concluded that COVID-19-related anxiety levels among pregnant women are similar in many countries. Another Turkish study found that 27.6% of pregnant women described COVID-19 anxiety as “moderate”. Furthermore, 52.6% of women expressed their willingness to receive COVID-19 vaccine when it was available [40]. During the COVID-19 pandemic, Berthelot et al. showed that pregnant women had higher levels of stress, anxiety and depression compared to pregnant women surveyed before the pandemic [41]. Durankus et al. showed that more than ^1^/_3_ of pregnant women experienced symptoms of depression and anxiety during the COVID-19 pandemic [42], which also confirms the results obtained in our study. Esteban-Gonzalo et al. reported that the level of concern about COVID-19-related symptoms and disease complications, infection and consequences for the child, restrictive measures and isolation due to COVID-19, delivery, postpartum and breastfeeding were also associated with higher levels of anxiety in pregnant women [43]. 

Our study found that pregnant women showed a relatively high acceptance of COVID-19 vaccination, with 51% of pregnant women saying that they would get vaccinated, but only 25% of the pregnant women surveyed commented that COVID-19 vaccination “is safe and necessary”. Different results were obtained by Ayhan et al., who conducted their study in a population of pregnant women in Turkey. In this study, only 37% of women were willing to receive the vaccine [44]. In the same Turkish study, among women who refused the vaccine, up to 66% cited lack of safety data on COVID-19 vaccine in pregnant women as the reason, whereas 42% of women claimed that the vaccine would harm their unborn child [44]. A similar response on the occurrence of defects in the child was chosen by 20% of the pregnant women surveyed in our study. Pregnant women in the Czech Republic also showed acceptance of COVID-19 vaccine (up to 76.6%), with the safety of vaccines for their unborn children being their highest priority [45]. Tao et al. found that the level of acceptance of COVID-19 vaccine among Chinese pregnant women in November 2020 was 77.4%, with the insufficiently defined safety of the vaccine being the most important determinant of vaccine hesitancy [46]. Gancer et al. found that 29.6% of pregnant women in Turkey showed a reluctant attitude to vaccine. A belief that vaccines were not safe, concern about adverse effects and exposure to negative news from the media or the Internet were the most important reasons for vaccine hesitancy [40]. Studies in Malaysia, Turkey and Canada found that lack of confidence and fear of adverse effects were the most important reasons for vaccine hesitancy [47,48,49]. Those who experienced hesitancy were convinced that vaccination was associated with a higher risk than the infection itself [48]. Gencer et al. reported that the pandemic had a positive impact on pregnant women’s decisions to vaccinate themselves and their children in the future [40].

In our study, the trimester of pregnancy was associated with an increase in coronavirus anxiety. The level of anxiety measured with FCV-19S increased with the trimester of pregnancy. In our study, the probability of receiving COVID-19 vaccine as measured with DrVac-COVID19S and VAC-COVID-19 was at a similar level in each trimester of pregnancy. Other studies demonstrated the highest levels of acceptance in pregnant women in the third trimester. A number of vaccines, including influenza vaccines, are recommended particularly in the third trimester in many countries. It can be assumed that vaccination in the third trimester is widely accepted by both pregnant women and the rest of society [38,50,51,52]. Therefore, it is understandable that pregnant women avoid medications in the first trimester, which is closely related to the period of organogenesis taking place during this period of pregnancy [53].

Pain at the injection site was the most common vaccine-induced complication in the study group (52%). The same symptom occurred in 72% of controls. Influenza-like symptoms such as fever, chills and muscle pain occurred in 33% of pregnant women and 47% of controls. Baden et al. also found pain and oedema at the injection site and influenza-like symptoms to be the most common vaccine-induced complications [54]. Sadoff et al. described a study conducted in eight countries on three continents. Their data showed that pain at the injection site (48.6%), headache (38.9%), fatigue (38.2%) and muscle pain (33.2%) were the most common complications after receiving COVID-19 vaccine [55]. The European Medicines Agency (EMA) also reports that pain and tenderness at the injection site, headache, fatigue, myalgia, general malaise, chills, fever, arthralgia and nausea were the most common symptoms [56].

Our analysis shows that mRNA vaccine was the most preferred preparation in both study (66%) and control group (75%). Rzymski et al. also reported that the Polish population most frequently vaccinated with mRNA preparations [57]. Similarly, pregnant women from the Czech Republic, when asked about their preferred COVID-19 vaccine type, confirmed their confidence in mRNA-based vaccines to be as high as 58.6% [45]. 

The results of our study suggest that foetal safety of the unborn child is a major factor influencing pregnant women’s decisions to receive the COVID-19 vaccine. The failure to include pregnant women in the COVID-19 vaccine study does not help with decision-making. Women should be supported with reliable information from trusted midwives and gynaecologists so that they can make an informed choice, guided by expert interpretation of the available data.

### Limitations

This study has certain limitations. The presented results were obtained in a study based on a subjective assessment of feelings and symptoms of anxiety occurring in pregnant women and women of reproductive age. The study used standardised scales, which are sensitive research tools, but their assessment relies on subjective feelings and does not include objective criteria for clinical symptoms, which may contribute to false-positive results. Sample size is another limitation of the study. The small group included in the study does not allow the results to be generalised to the entire population of pregnant women in Poland. However, in spite of the limitations, the results presented may be a reference point for further research on the level of fear of COVID-19 and attitudes towards COVID-19 vaccines among women both in Poland and worldwide.

## 5. Conclusions

Pregnant women generally showed moderate COVID-19 anxiety, but the results varied depending on the tool used. VAC-COVID-19 and DrVac-COVID19S scores confirmed the high level of vaccine acceptance among the women surveyed and positive attitudes towards it. There was a strong positive correlation between VAC-COVID-19 and DrVac-COVID19S. Insufficient knowledge of the effects or complications of the vaccine in the foetus were the most common reason for COVID-19 vaccine refusal among pregnant women. The availability of accurate information can positively influence vaccination rates in this population. The results for this particular sample are linked to the results of national and international studies. It is advisable to develop new training projects for healthcare system employees, in particular in the field of perinatal care, in order to better identify and take into account COVID-19 stressors and provide the best psychological support based on the needs and requirements related to this challenge, as well as apply a system of incentives for vaccination against COVID-19, taking into account the benefits for both expectant mothers and their unborn children. Moreover, the topic should be explored in more detail, to this end by carrying out longitudinal studies. 

## Figures and Tables

**Table 1:** Socio-demographic characteristics of respondents.

Socio-Demographic Characteristics		Study Group		Control Group	
		*n*	%	*n*	%
Education	primary	1	0%	5	2%
	middle school	9	3%	5	2%
	basic vocational	18	6%	25	8%
	secondary	126	44%	116	38%
	higher	134	47%	156	51%
Marital status	married	235	82%	153	50%
	divorced	9	3%	15	5%
	single	37	13%	113	37%
	in an informal relationship	7	2%	23	8%
Place of residence	urban	177	61%	218	71%
	rural	111	39%	89	29%
Socioeconomic status	very good	47	16%	59	19%
	good	179	62%	189	61%
	average	62	22%	59	19%
Number of children	0	139	48%	151	49
	1	88	30.5%	48	16
	2	33	11.5%	77	25
	3	23	8%	23	7.5
	4	4	1.3%	7	2.3
	5	1	0.3%	0	0
	8	0	0%	1	0.3

**Table 2:** Analysis of the author’s original questionnaire.

Question		Study Group		Control Group	
		*n*	%	*n*	%
Are you vaccinated against COVID-19?	yes	147	51	222	72
	no	141	49	85	28
Did you suffer from COVID-19?	yes	51	18	150	49
	no	237	82	157	51
What vaccine have you received?	Pfizer	97	66	167	75
	Moderna	21	14	25	11
	Astra-Zeneca	25	17	24	11
	Johnson & Johnson	10	7	18	8
Symptoms after vaccination	pain at the injection site	77	52	160	72
	swelling/local inflammation at injection site	10	7	26	12
	flu-like symptoms: fever, chills, muscle pain	48	33	104	47
	Runny nose, cough	6	4	21	9
	anaphylactic reaction	0	0	0	0
	no symptoms have occurred	49	33	41	18
What is your opinion on vaccinating pregnant women against COVID-19?	it is safe and necessary	72	25	128	42
	it is safe, but I don’t want to be vaccinated	22	8	12	4
	vaccination will shorten the pandemic period	19	7	46	15
	it is dangerous	29	10	23	8
	it is contraindicated in pregnancy	14	5	20	7
	I have no opinion	142	49	115	38
What do you think are the effects of the COVID-19 vaccination?	acquisition of specific immunity against COVID-19 by both mother and child	127	44	154	50
	acquisition of specific immunity against COVID-19 by the mother	24	8	59	19
	acquisition of specific immunity against COVID-19 by the child	9	3	10	3
	preterm birth	28	10	17	6
	defects in the child	59	20	38	12
	perinatal complications (obstetric haemorrhage, etc.)	32	11	19	6
	infertility or problems getting pregnant in the future	48	17	31	10
	mutations or changes in the karyotype (genetic code) of the pregnant woman	17	6	25	8

**Table 3:** Summary of COVID-19 rates and vaccine coverage.

Have You Had COVID-19?		Are You Vaccinated against COVID-19?			
		Study Group		Control Group	
		Yes	No	Yes	No
yes	*n*	18	33	109	41
	%	12	23	49	48
no	*n*	129	108	113	44
	%	88	77	51	52

**Table 4:** Descriptive statistics of standardised scales.

Scales	Control Group (*n* = 307)					Study Group (*n* = 288)					*p*
	M	Sd	Q_1_	Me	Q_3_	M	Sd	Q_1_	Me	Q_3_	
CAS	1.03	1.98	0	0	1	0.61	1.26	0	1.26	1	0.025
DrVac-COVID19S	55.85	14.41	45	57	68	51.25	13.97	43	49.5	62	0.000
FCV-19S	15.21	4.93	12	15	18	14.59	5.26	11	14	18	0.126
VAC-COVID-19	44.26	7.21	39	45	50	41.44	7.27	36	41	47	0.000

Abbreviations: CAS—Coronavirus Anxiety Scale, FCV-19S—The Fear of COVID-19 Scale, M—mean, Me—median, *p—p*-value, SD—standard deviation, Q_1_—lower quartile, and Q_3_—upper quartile.

**Table 5:** Correlations between scores and pregnancy trimester.

Scales	Study Group-Pregnant Women								
	1st Trimester (*n* = 43)			2nd Trimester (*n* = 92)			3rd Trimester (*n* = 153)		
	M	Sd	Me	M	Sd	Me	M	Sd	Me
CAS	0.67	1.36	0	0.61	1.44	0	0.59	1.12	0
DrVac-COVID19S	53.86	13.67	51	51.25	13.21	49	50.52	14.50	49
FCV-19S	12.34	4.94	12	14.70	5.20	15	15.16	5.25	15
VAC-COVID-19 Scale	43.00	8.00	44	41.45	7.30	41	41.00	7.03	40

Abbreviations: CAS—Coronavirus Anxiety Scale, FCV-19S—The Fear of COVID-19 Scale, M—mean, Me—median, SD—standard deviation.

**Table 6:** Inter-scale correlations by groups.

		Spearman’s r	
		Control Group (*n* = 307)	Study Group (*n* = 288)
CAS	DrVac-COVID19S	0.053	−0.005
	FCV-19S	0.377	0.364
DrVac-COVID19S	FCV-19S	0.207	0.196
VAC-COVID-19 Scale	CAS	0.108	0.020
	DrVac-COVID19S	0.722	0.753
	FCV-19S	0.178	0.082

Abbreviations: CAS—Coronavirus Anxiety Scale, FCV-19S—The Fear of COVID-19 Scale.

## Data Availability

Data are available upon reasonable request.
